# Precision Medicine for Treating Alzheimer’s Disease in Down Syndrome

**DOI:** 10.1002/alz.093181

**Published:** 2025-01-09

**Authors:** Sonal Sukreet, Elise K Kim, Melissa Petersen, Fan Zhang, Mary Sano, Paul S. S. Aisen, Howard F Andrews, Sid E. O'Bryant, Michael Rafii, Robert A. Rissman

**Affiliations:** ^1^ University of California, San Diego, La Jolla, CA USA; ^2^ University of North Texas Health Science Center, Fort Worth, TX USA; ^3^ Icahn School of Medicine at Mount Sinai, New York, NY USA; ^4^ Alzheimer's Therapeutic Research Institute, Keck School of Medicine, University of Southern California, San Diego, CA USA; ^5^ Columbia University, New York, NY USA; ^6^ Alzheimer's Therapeutic Research Institute, University of Southern California, San Diego, CA USA; ^7^ Department of Neurosciences, University of California San Diego, La Jolla, CA USA

## Abstract

**Background:**

Individuals with Down Syndrome (DS) exhibit a genetic form of Alzheimer's disease (AD). In this study, we leveraged biobanked plasma samples from a completed clinical trial focused on anti‐inflammatory treatment, Vitamin E, for DS. The trial's primary endpoint was on cognition, and Vitamin E treatment was not found to be significantly beneficial. We examined whether applying precision medicine using an established proinflammatory endophenotype algorithm for AD therapy would reveal responders to anti‐inflammatory therapy as we have with other AD within the DS trial sample.

**Method:**

We utilized biobanked plasma samples from the Phase 3 clinical trial titled "Vitamin E in Aged Persons with Down Syndrome (NCT00056329)." A pro‐inflammatory panel of proteins, including pre‐defined predictive protein and neuropathology markers, were assayed in baseline (n=138) and 36‐month (n=138) plasma samples. Response status was determined by evaluating the annual rate of change as measured by a combination of primary and secondary endpoints (BPT, VT, and BFDS). The predictive biomarker profile was established through SVM analyses to predict treatment response (yes/no) as the outcome variable.

**Result:**

Pro‐inflammatory biomarkers IFN, IL10, IL12p70, 1L13, IL1B, IL2, IL4, IL6, IL8, and TNF𝛼 (along with APOE4 and demographic factors) were highly accurate in predicting the treatment responders with an AUC of 99.41%. Neuropathology markers, including Aβ40, Aβ42, Aβ42/Aβ40 ratio, NfL, GFAP, total tau, pTau181, and pTau231 with added demographic and APOE factors, predicted the responder status with an AUC of 73.98%. When the inflammatory and neuropathological data were combined into one algorithm, it produced an AUC of 97.35% (sensitivity of 81.69%, specificity of 100%) with an optimized cut‐off of 0.442 for distinguishing responder status.

**Conclusion:**

Our findings confirmed the utility of our established AD pro‐inflammatory endophenotype for use in the DS population. We demonstrate that a protein algorithm encompassing markers related to inflammation and neuropathology can be used to generate a predictive biomarker endophenotype that can identify DS participants who are more likely to respond and benefit from anti‐inflammatory treatment. Our results demonstrate the feasibility of using precision medicine approaches for treating AD in DS. Our ongoing studies focus on understanding how these predictive algorithms relate to neuropathological progression in DS.

